# Supplementary material for: Ecological Niche Modeling of Aedes and Culex Mosquitoes: A Risk Map for Chikungunya and West Nile Viruses in Zambia
Source: Viruses. 2023 Sep 8;15(9):1900. doi: 10.3390/v15091900 (PMC10535978; doi:10.3390/v15091900)
Supplement: Supplementary file 1 [file viruses-15-01900-s001.zip › Table S1. Aedes occurrence data.pdf]

species	longitude	latitude
Aedes	28.31193	-15.3715
Aedes	28.27267	-15.3595
Aedes	27.4791	-17.2224
Aedes	28.46043	-14.4207
Aedes	28.1947	-15.2319
Aedes	31.1103	-8.7692
Aedes	32.3839	-10.0919
Aedes	31.2702	-10.4841
Aedes	32.3839	-13.3833
Aedes	24.2553	-11.4308
Aedes	23.1306	-15.254
Aedes	24.1743	-17.2757
Aedes	25.1632	-17.4623
Aedes	25.4832	-17.5106
Aedes	28.5119	-16.0141
Aedes	29.1316	-15.1903
Aedes	24.4759	-13.4103
Aedes	24.1227	-13.3548
Aedes	22.414	-13.0513
Aedes	24.4749	-14.4839
