# Supplementary material for: Ecological Niche Modeling of Aedes and Culex Mosquitoes: A Risk Map for Chikungunya and West Nile Viruses in Zambia
Source: Viruses. 2023 Sep 8;15(9):1900. doi: 10.3390/v15091900 (PMC10535978; doi:10.3390/v15091900)
Supplement: Supplementary file 1 [file viruses-15-01900-s001.zip › Table S2. Culex occurrence data.pdf]

species	longitude	latitude
Culex	28.29819	-15.3815
Culex	28.2932	-15.3759
Culex	28.31437	-15.3687
Culex	28.31193	-15.3715
Culex	28.30455	-15.3764
Culex	28.29384	-15.3763
Culex	27.4791	-17.2224
Culex	28.28	-15.4589
Culex	28.27267	-15.3595
Culex	28.27573	-15.4579
Culex	28.27914	-15.4594
Culex	28.29738	-15.3751
Culex	28.46043	-14.4207
Culex	28.1428	-15.232
Culex	28.1808	-15.2042
Culex	28.1948	-15.2418
Culex	28.2217	-15.2127
Culex	28.0624	-15.3024
Culex	31.1103	-8.7692
Culex	32.3839	-10.0919
Culex	31.2702	-10.4841
Culex	32.3839	-13.3833
Culex	27.5141	-12.3251
Culex	28.1257	-12.4947
Culex	24.1873	-11.608
Culex	23.1306	-15.264
Culex	24.1743	-17.2757
Culex	25.1632	-17.4623
Culex	25.4832	-17.1506
Culex	28.5119	-16.0141
Culex	29.1316	-15.1903
Culex	26.2551	-15.445
Culex	27.4452	-15.5135
Culex	28.4321	-16.3213
Culex	22.4035	-14.593
