# Supplementary material for: Ecological Niche Modeling of Aedes and Culex Mosquitoes: A Risk Map for Chikungunya and West Nile Viruses in Zambia
Source: Viruses. 2023 Sep 8;15(9):1900. doi: 10.3390/v15091900 (PMC10535978; doi:10.3390/v15091900)
Supplement: Supplementary file 1 [file viruses-15-01900-s001.zip › Table S3. Multicollinearity test of environmental variables.pdf]

	bio_1	bio_2	bio_3	bio_4	bio_5	bio_6	bio_7	bio_8	bio_9	bio_10	bio_11	bio_12	bio_13	bio_14	bio_15	bio_16	bio_17	bio_18	bio_19	elev_1
bio_1	1	-0.12056	0.166448	-0.08404	0.734349	0.713034	-0.1377	0.902399	0.854774	0.886557	0.847543	-0.20411	-0.30805	0.156766	-0.05677	-0.36711	0.18117	-0.15538	0.10794	-0.83187
bio_2	-0.12056	1	-0.31682	0.567237	0.436297	-0.70482	0.889836	0.055428	-0.39633	0.083639	-0.39024	-0.38352	-0.35218	-0.25819	0.288649	-0.28511	-0.334	-0.20078	-0.13771	0.007848
bio_3	0.166448	-0.31682	1	-0.86675	-0.3489	0.527258	-0.68079	-0.10124	0.525726	-0.20896	0.565255	0.588347	0.172674	0.153348	-0.78091	0.117645	0.452199	0.039564	0.281083	0.111969
bio_4	-0.08404	0.567237	-0.86675	1	0.47455	-0.60651	0.83101	0.255886	-0.52002	0.32279	-0.56627	-0.79345	-0.41179	-0.08631	0.828294	-0.36769	-0.38388	-0.0454	-0.23405	-0.20191
bio_5	0.734349	0.436297	-0.3489	0.47455	1	0.139274	0.517555	0.828035	0.393049	0.889205	0.37223	-0.54432	-0.45667	-0.01181	0.374288	-0.45081	-0.12895	-0.27443	-0.05387	-0.74827
bio_6	0.713034	-0.70482	0.527258	-0.60651	0.139274	1	-0.76339	0.487967	0.901767	0.450618	0.896743	0.261755	0.092486	0.305012	-0.38308	0.010926	0.428701	0.008667	0.215693	-0.49243
bio_7	-0.1377	0.889836	-0.68079	0.83101	0.517555	-0.76339	1	0.116969	-0.52183	0.188542	-0.53134	-0.57837	-0.37569	-0.26817	0.571972	-0.30215	-0.45091	-0.18418	-0.21871	-0.06169
bio_8	0.902399	0.055428	-0.10124	0.255886	0.828035	0.487967	0.116969	1	0.644175	0.945222	0.617086	-0.47827	-0.46402	0.168823	0.203516	-0.5149	0.095286	-0.06825	0.027169	-0.88812
bio_9	0.854774	-0.39633	0.525726	-0.52002	0.393049	0.901767	-0.52183	0.644175	1	0.602585	0.974777	0.198821	-0.03168	0.273388	-0.41532	-0.11207	0.410585	-0.09049	0.231307	-0.62958
bio_10	0.886557	0.083639	-0.20896	0.32279	0.889205	0.450618	0.188542	0.945222	0.602585	1	0.575355	-0.51754	-0.43476	0.112792	0.313801	-0.4718	0.005133	-0.19671	0.004795	-0.85589
bio_11	0.847543	-0.39024	0.565255	-0.56627	0.37223	0.896743	-0.53134	0.617086	0.974777	0.575355	1	0.219994	-0.05323	0.171143	-0.45254	-0.12371	0.339906	-0.11068	0.20578	-0.58656
bio_12	-0.20411	-0.38352	0.588347	-0.79345	-0.54432	0.261755	-0.57837	-0.47827	0.198821	-0.51754	0.219994	1	0.791403	0.150664	-0.72325	0.767713	0.40181	0.183675	0.213925	0.331157
bio_13	-0.30805	-0.35218	0.172674	-0.41179	-0.45667	0.092486	-0.37569	-0.46402	-0.03168	-0.43476	-0.05323	0.791403	1	0.151207	-0.19162	0.970378	0.222292	0.185812	0.11738	0.277597
bio_14	0.156766	-0.25819	0.153348	-0.08631	-0.01181	0.305012	-0.26817	0.168823	0.273388	0.112792	0.171143	0.150664	0.151207	1	-0.20259	0.105144	0.8758	0.245972	0.329981	-0.26453
bio_15	-0.05677	0.288649	-0.78091	0.828294	0.374288	-0.38308	0.571972	0.203516	-0.41532	0.313801	-0.45254	-0.72325	-0.19162	-0.20259	1	-0.12916	-0.51142	-0.13171	-0.28321	-0.17683
bio_16	-0.36711	-0.28511	0.117645	-0.36769	-0.45081	0.010926	-0.30215	-0.5149	-0.11207	-0.4718	-0.12371	0.767713	0.970378	0.105144	-0.12916	1	0.15436	0.151821	0.070676	0.320999
bio_17	0.18117	-0.334	0.452199	-0.38388	-0.12895	0.428701	-0.45091	0.095286	0.410585	0.005133	0.339906	0.40181	0.222292	0.8758	-0.51142	0.15436	1	0.259054	0.414815	-0.20799
bio_18	-0.15538	-0.20078	0.039564	-0.0454	-0.27443	0.008667	-0.18418	-0.06825	-0.09049	-0.19671	-0.11068	0.183675	0.185812	0.245972	-0.13171	0.151821	0.259054	1	0.026923	0.014337
bio_19	0.10794	-0.13771	0.281083	-0.23405	-0.05387	0.215693	-0.21871	0.027169	0.231307	0.004795	0.20578	0.213925	0.11738	0.329981	-0.28321	0.070676	0.414815	0.026923	1	-0.08711
elev_1	-0.83187	0.007848	0.111969	-0.20191	-0.74827	-0.49243	-0.06169	-0.88812	-0.62958	-0.85589	-0.58656	0.331157	0.277597	-0.26453	-0.17683	0.320999	-0.20799	0.014337	-0.08711	1

Variable Description	Abbreviati	Unit
Annual Mean Temperature	BIO1	0c
Mean Diurnal Range (Mean of monthly (max temp - min temp))	BIO2	0c
Isothermality (BIO2/BIO7) (×100)	BIO3	0c
Temperature Seasonality (standard deviation ×100)	BIO4	0c
Max Temperature of Warmest Month	BIO5	0c
Min Temperature of Coldest Month	BIO6	0c
Temperature Annual Range (BIO5-BIO6)	BIO7	0c
Mean Temperature of Wettest Quarter	BIO8	0c
Mean Temperature of Driest Quarter	BIO9	0c
Mean Temperature of Warmest Quarter	BIO10	0c
Mean Temperature of Coldest Quarter	BIO11	0c
Annual Precipitation	BIO12	Mm
Precipitation of Wettest Month	BIO13	Mm
Precipitation of Driest Month	BIO14	Mm
Precipitation Seasonality (Coefficient of Variation)	BIO15	Mm
Precipitation of Wettest Quarter	BIO16	Mm
Precipitation of Driest Quarter	BIO17	Mm
Precipitation of Warmest Quarter	BIO18	Mm
Precipitation of Coldest Quarter	BIO19	Mm
